# The cerebellum: from development to structural complexity and motor learning

**DOI:** 10.3389/fnana.2014.00118

**Published:** 2014-11-28

**Authors:** Salvador Martinez

**Affiliations:** ^1^Developmental Biology Unit, Instituto Neurociecnias, University Miguel Hernandez-Consejo Superior de Investigaciones CientíficasAlicante, Spain; ^2^Brain Morphogenesis, Instituto Murciano de Investigación Biosanitaria-Arrixaca, University of MurciaMurcia, Spain

**Keywords:** cerebellum development, cerebellum and cognition, brain regionalization, Purkinje neuron, cerebellar regionalization, cerebellum circuits, opto-kinetic reflex, cerebellar interneurons

The cerebellum coordinates motor activities to be performed or that are already underway. In fact, it is very well known that cerebellar damage produces disturbance in movements and in body support. The relationship between cerebellum and motor learning was first suggested with the studies of Ramón y Cajal (1911), Dow and Moruzzi (1958), and Eccles et al. (1967). Dow and Moruzzi (1958) hypothesized that the cerebellum contributes to motor learning by determining how to perform accurate and correct movements (revised in Ito, 2002). Thereafter, numerous studies have been devoted to analyzing the role of the cerebellum in perceptive and cognitive processes. Thus, the essential contribution of Marr, localizing the site of motor learning in the cerebellar cortex (Marr, 1969), and the later application of Marr's theory to the classical conditioning (Albus, 1971), whose physiological basis is directly related to long-term depres-sion (LTD) mechanisms (Ito, 1989), defined the neuronal circuit involved in associative motor learning (revised by Porras-García et al., 2013). Although this appearance of agreement in these general aspects of cerebellar contribution to motor learning, the underlying cellular mechanisms of this contribution are far to be clear. Actually, recent reviews were published trying to get a consensus about the functional complexity that links the cerebellar intrinsic and extrinsic circuits with the motor coordination and learning; to conclude that there is still a lot of work to be done to get a precise idea about cerebellar cognitive function and the physiopathology of behavioral deficits in cerebellar dysfunctions (Ito, 2006, 2008; Manto et al., 2012). In this book, functional mechanisms of cerebellar circuits involved in motor learning are represented by the contribution of Dr. Jose Maria Delgado-Garcia's group (Sánchez-Campusano et al., 2012), where by modern electrophysiological methods they accurately analyzed the role of interpositus neurons in eyelid kinetics, demonstrating that antagonistic groups of deep cerebellar nuclei neurons are required for proper dynamic control of learned motor responses.

In parallel to the knowledge deficits on cerebellar function, cerebellar morphogenesis, which seems to be simple due to the repetitive myelo- and cyto-architecture along the whole organ, has revealed in the last two decades inspected complexities. First by the discovering of molecular heterogeneities in Purkinje cells; that is, new antigens (among then zebrins were pioneers) revealed a repetitive-stripe like structural organization of cerebellar Purkinje cells that reminded sagittal distribution of cortical afferences and efferences in the cerebellum. Then, probably for first time in vertebrates the molecular architecture reminded functional organization of neuronal circuits. Moreover, the expression patterns of these markers during development showed how Purkinje cells regulate the fundamental processes in the structural and functional development of the whole cerebellum. This was a seminal result to develop causal ontogenetic studies on the molecular control of cerebellar structure and function (reviewed in Dastjerdi et al., 2012; White and Sillitoe, 2013). Second, complexity also derived from experimental embryology approaches revealed a heterogeneous origin of cerebellar precursors, from at least three different domains: caudal mesencephalon, isthmus and rhombencephalon. Each domain originated different cerebellar regions: anterior and posterior vermis, as well as the cerebellar hemispheres, respectively (Martinez et al., 2013). Moreover we have strongly advanced in identifying the molecular mechanisms underlying internal cerebellar regionalization. The revisions from Martinez et al. (2013) and Basson and Wingate (2013) describe the embryology and morphogenesis of cerebellar anlage, which is controlled by different organizer regions and morphogenetic signals. The relation between neuroepithelial microdomains and Purkinje neurons specification to develop antigenic-defined stripes is extensively revised in Dastjerdi et al. (2012).

The contribution from Dr. Ferdinando Rossi laboratory (Leto et al., 2012) describes how neural progenitors of cerebellar GABAergic neurons have different origin, in relation to the neuronal character: (1) projection GABAergic neurons are originated from ventricular progenitors locally committed to their fate under cell autonomous mechanisms (Leto et al., 2012); which are consequence of positional information defined microdomains in the neuroepithelium (see Dastjerdi et al., 2012; Basson and Wingate, 2013; Martinez et al., 2013). (2) Conversely, GABAergic cerebellar interneuron progenitors are multipotent and sensitive to spatio-temporally patterned environmental signals that regulate the genesis of different categories of interneurons, in precise quantities and at defined times and places. Our friend Dr. Ferdinando Rossi passed away on January 24th, 2014, shortly after having made this contribution. His excellent and highly significant scientific legacy will continue to illuminate us in understanding the cerebellum.

Dr. Isabelle Dusart's group contribution (Dusart and Flamant, 2012) describes the strong structural changes that Purkinje neurons suffer during the two first postnatal weeks and the significant role of thyroid hormones in this process. The cerebellar alterations of hypothyroidism have been described in Dr. Manto and Dr. Jissendi paper (2913).

Barkovich (2012) revises the most frequent malformation patterns in humans and discusses about potential underling causal molecular and cellular mechanisms that operating during cerebellar development can explain the observed malformations. This revision is complemented by the contribution of Manto and Jissendi (2012) were cerebellar anomalies associated to genes regulating neural migration and synaptogenesis were revised, together with other noxious situations. Interestingly, pathogenic predictions developed from the molecular and genetic embryonic approaches (Basson and Wingate, 2013; Martinez et al., 2013) were clearly recognizable in the described clinical phenotypes.

## Conflict of interest statement

The author declares that the research was conducted in the absence of any commercial or financial relationships that could be construed as a potential conflict of interest.

## References

[B1] Albus J. S. (1971). A theory of cerebellar function. Math. Biosci. 10, 25–61.

[B2] Barkovich A. J. (2012). Developmental disorders of the midbrain and hindbrain. Front. Neuroanat. 6:7. 10.3389/fnana.2012.00007 22408608 PMC3294267

[B3] Basson M. A. Wingate R. J. (2013). Congenital hypoplasia of the cerebellum: developmental causes and behavioral consequences. Front. Neuroanat. 7:29. 10.3389/fnana.2013.00029 24027500 PMC3759752

[B4] Dastjerdi F. V. Consalez G. G. Hawkes R. (2012). Pattern formation during development of the embryonic cerebellum. Fornt. Neuronat. 6:10. 10.3389/fnana.2012.00010 22493569 PMC3318227

[B5] Dow R. E. Moruzzi G. (1958). The Physiology and Pathology of the Cerebellum. Minneapolis, MN: University of Minnesota Press.

[B6] Dusart I. Flamant F. (2012). Profound morphological and functional changes of rodent Purkinje cells between the first and the second postnatal weeks: a metamorphosis? Front. Neuroanat. 6:11. 10.3389/fnana.2012.00011 22514522 PMC3324107

[B7] Eccles J. C. Ito M. Szentagothai J. (1967). The Cerebellum as a Neuronal Machine. New York, NY: Springer-Verlag.

[B8] Ito M. (1989). Long-term depression. Annu. Rev. Neurosci. 12, 85–102. 2648961 10.1146/annurev.ne.12.030189.000505

[B9] Ito M. (2002). Historical Review of the significance of the cerebellum and the role of Purkinje cells in motor learning. Ann. N.Y. Acad. Sci. 978, 273–288. 10.1111/j.1749-6632.2002.tb07574.x 12582060

[B9a] Ito M. (2006). Cerebellar circuitry as a neuronal machine. Prog. Neurobiol. 78, 272–303. 10.1016/j.pneurobio.2006.02.006 16759785

[B10a] Ito M. (2008). Control of mental activities by internal models in the cerebellum. Nat. Rev. Neurosci. 9, 304–313. 10.1038/nrn2332 18319727

[B18] Leto K. Rolando C. Rossi F. (2012). The genesis of cerebellar GABAergic neurons: fate potential and specification mechanisms. Front. Neuronat. 6:6. 10.3389/fnana.2012.00006 22363268 PMC3282257

[B11] Manto M. Bower J. M. Conforto A. B. Delgado-García J. M. da Guarda S. N. Gerwig M.. (2012). Consensus paper: roles of the cerebellum in motor control—the diversity of ideas on cerebellar involvement in movement. Cerebellum 11, 457–487. 10.1007/s12311-011-0331-9 22161499 PMC4347949

[B11a] Manto M. U. Jissendi P. (2012). Cerebellum links between development, developmental disorders and motor learning. Front. Neuroanat. 6:1. 10.3389/fnana.2012.00001 22291620 PMC3263706

[B12] Marr D. (1969). A theory of cerebellar cortex. J. Physiol. 202, 437–470. 5784296 10.1113/jphysiol.1969.sp008820 PMC1351491

[B13] Martinez S. Andreu A. Mecklenburg N. Echevarria D. (2013). Cellular and molecular basis of cerebellar development. Front. Neuroanat. 7:18. 10.3389/fnana.2013.00018 23805080 PMC3693072

[B14] Porras-García M. E. Ruiz R. Pérez-Villegas E. M. Armengol J. A. (2013). Motor learning of mice lacking cerebellar Purkinje cells. Fornt. Neuroanat. 7:4. 10.3389/fnana.2013.00004 23630472 PMC3632800

[B15] Ramón y Cajal S. (1911). Histologie du Systeme Nerveus de l'home et des Vertevres. Paris: Maloine.

[B16] Sánchez-Campusano R. Gruart A. Fernández-Mas R. Delgado-García J. M. (2012). An agonist–antagonist cerebellar nuclear system controlling eyelid kinematics during motor learning. Front. Neuroanat. 6:8. 10.3389/fnana.2012.00008 22435053 PMC3303085

[B17] White J. J. Sillitoe R. V. (2013). Pastnatal development of cerebellar zones revealed by neirofilament heavy chain protein expression. Front. Neuroanat. 7:9. 10.3389/fnana.2013.00009 23675325 PMC3648691

